# Behavioral heterogeneity in quorum sensing can stabilize social cooperation in microbial populations

**DOI:** 10.1186/s12915-019-0639-3

**Published:** 2019-03-06

**Authors:** Kelei Zhao, Linjie Liu, Xiaojie Chen, Ting Huang, Lianming Du, Jiafu Lin, Yang Yuan, Yingshun Zhou, Bisong Yue, Kun Wei, Yiwen Chu

**Affiliations:** 1 Antibiotics Research and Re-evaluation Key Laboratory of Sichuan Province, Sichuan Industrial Institute of Antibiotics, Chengdu University, No. 168, Huaguan Road, Chengdu, 610052 Sichuan China; 2 School of Mathematical Sciences, University of Electronic Science and Technology of China, No. 2006, Xiyuan Avenue, Chengdu, 611731 Sichuan China; 3 Key Laboratory of Bio-resources and Eco-environment (Ministry of Education), College of Life Sciences, Sichuan University, Chengdu, China; 4 Institute for Advanced Study, Chengdu University, Chengdu, China; 5 Department of Pathogenic Biology, College of Preclinical Medicine, Southwest Medical University, Luzhou, China

**Keywords:** Social biology, Quorum sensing, Public goods game, Evolution, Conditional defection, Mathematical modeling

## Abstract

**Background:**

Microbial communities are susceptible to the public goods dilemma, whereby individuals can gain an advantage within a group by utilizing, but not sharing the cost of producing, public goods. In bacteria, the development of quorum sensing (QS) can establish a cooperation system in a population by coordinating the production of costly and sharable extracellular products (public goods). Cooperators with intact QS system and robust ability in producing public goods are vulnerable to being undermined by QS-deficient defectors that escape from QS but benefit from the cooperation of others. Although microorganisms have evolved several mechanisms to resist cheating invasion in the public goods game, it is not clear why cooperators frequently coexist with defectors and how they form a relatively stable equilibrium during evolution.

**Results:**

We show that in *Pseudomonas aeruginosa*, QS-directed social cooperation can select a conditional defection strategy prior to the emergence of QS-mutant defectors, depending on resource availability. Conditional defectors represent a QS-inactive state of wild type (cooperator) individual and can invade QS-activated cooperators by adopting a cheating strategy, and then revert to cooperating when there are abundant nutrient supplies irrespective of the exploitation of QS-mutant defector. Our mathematical modeling further demonstrates that the incorporation of conditional defection strategy into the framework of iterated public goods game with sound punishment mechanism can lead to the coexistence of cooperator, conditional defector, and defector in a rock-paper-scissors dynamics.

**Conclusions:**

These findings highlight the importance of behavioral heterogeneity in stabilizing the population structure and provide a potential reasonable explanation for the maintenance and evolution of cooperation in microbial communities.

**Electronic supplementary material:**

The online version of this article (10.1186/s12915-019-0639-3) contains supplementary material, which is available to authorized users.

## Background

Cooperation and defection are the most common strategies in the populations ranging from single-celled organisms to complex animals [1–4]. A behavior is cooperative if the produced public goods can bring benefits to the recipient, and such cooperative interaction is susceptible to being undermined by selfish behaviors (cheating) and may cause the “tragedy of the commons” [5–7]. Explaining the evolution and maintenance of cooperation in the public goods game in which defection is the stable state brings a significant challenge for biologists and sociologists, and numerous excellent theoretical and experimental analyses have developed a series of frameworks to explain the emergence of cooperative behaviors [8–10].

**Fig. 1:**
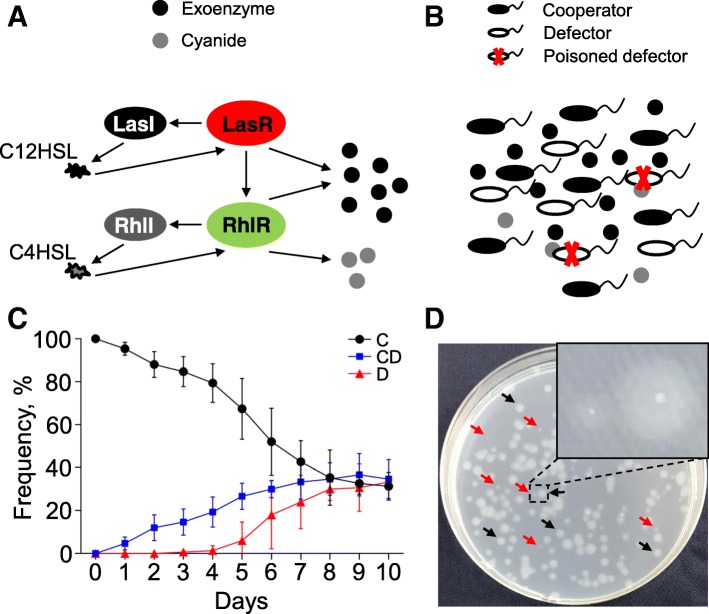
Cell-cell interaction and social structure of *P. aeruginosa* during evolution. **a** Paradigm of *P. aeruginosa* QS system and **b** the scenario of bacterial public goods game. LasR sits in the central of QS hierarchy can activate the production of exoenzymes (public goods), which is costly to producers but can be freely used by individuals in the pool. The RhlRI circuit can be triggered by the LasR-C12HSL complex and governs the secretion of other public goods including cyanide. WT cells with robust QS activity are defined as cooperators, and the *lasR* mutants act as defectors can exploit the public goods but may suffer the cyanide-mediated social punishment. **c** Population divergence of repeatedly subcultured WT PAO1 in 1.0 ml of M9-casein (0.5%) broth at 24-h intervals. Data shown are the mean values ± SD of three independent experiments. **d** Phenotypic heterogeneity of repeatedly subcultured WT PAO1 in QSM. A fraction of culture liquid from day 3 of the in vitro evolution assay was spread on M9-milk plate after gradient dilutions and cultured for 12 h. Black arrows indicate the colony with proteolysis halo; red arrow indicates the slow-growing colony with no apparent proteolysis halo. C, cooperator; CD, conditional defector; D, defector

Microbial cell-cell interactions can work as an ideal paradigm to study the evolutionary dynamics of social cooperation [11–15]. The notorious pathogen *Pseudomonas aeruginosa*, which normally causes a variety of acute and chronic infections, is a good model for studying microbial public goods game [3, 16]. The cooperative interactions of *P. aeruginosa* are mainly controlled by the elaborate quorum sensing (QS) system, which regulates the production of most extracellular factors (such as proteases, virulence factors, and signal molecules) for population fitness in diverse environments [16–18]. The QS-mediated social cooperation in *P. aeruginosa* has been well-characterized (Fig. 1a, b): LasR acting as the central regulator of QS hierarchy can receipt the accumulated autoinducer (*N*-3-oxo-dodecanoyl-homoserine lactone, C12HSL) synthesized by LasI and triggers the expression of a variety of public-goods-encoding genes. The activated LasR can also promote the expression of *lasI* and thus set up the positive feedback loop. The subordinate QS circuit RhlR-RhlI, which is triggered by LasR-C12HSL complex, can activate the production of other sets of public goods including cyanide in dependence on the synthesis of another autoinducer (*N*-butanoyl-homoserine lactone, C4HSL) [17, 18]. The wild type individuals with activated QS regulation are defined as cooperators, and their cooperation is vulnerable to invasion by the typical *lasR* mutant defectors that escape from QS but still benefit from the cooperative behaviors of others, thereby indicating the scenario of public goods game in bacterial population during evolution [19–23]. Moreover, like human society, *P. aeruginosa* also evolves the ability of within-group punishment by using cooperator-released toxic cyanide to exclude *lasR* mutant defectors which lack the LasR regulon for detoxification [23–25].

Based on the currently known relationship of cooperation and defection in *P. aeruginosa* (Fig. 1b), we first develop a mathematical model to predict their evolutionary dynamics in a well-mixed population using evolutionary game theory. However, the scenario of coordination game [26], in which the ultimate winner is dependent on the initial frequencies of strategies and the degree of punishment, is obtained (Additional file 1: Figure S1). This is incongruent with the fact that a relatively stable equilibrium of cooperators and defectors frequently forms during the in vitro evolution of *P. aeruginosa* [19, 22, 23]. A possible explanation is that unlike the fixed character setup of each player in mathematical modeling, bacteria evolve several capabilities to adapt to a variety of natural or unnatural niches by deploying versatile intracellular regulation rather than depending on a monotonous strategy or mutagenesis [18, 27–30]. Fundamentally, the nomenclature of “quorum” in QS is introduced to describe the minimum quantity of local bacterial cells to efficiently express a large number of genes in the genome [31, 32]. This process is energy-demanding and requires the support of sufficient resource supplies [33, 34]. There are evidences that the production of quorum-controlled public goods in microbial populations will be reduced during evolution, and an optimal mixture of cooperative and cheating behaviors can maximize population size [34–37]. Furthermore, although it is well accepted that QS brings more benefits at higher cell densities [38], the expression of quorum-controlled genes are found to be triggered by specific class of environmental cues but unrelated to cell density [33, 39], and not every individual in a stationary population will have the opportunity to receipt the QS signals [40, 41]. Therefore, the environment-dependent activation of QS suggests that a pervasive cooperative interaction may not always be required during bacterial growth, and strongly implies the existence of conditional strategy in bacterial public goods game.

In this study, we reproduce a series of in vitro evolution assays of *P. aeruginosa* and find that in addition to the QS mutants with heritable defection strategy, bacterial social cooperation also creates selection for conditional defectors which show a cheating phenotype but have the potential to cooperate according to the change of growth conditions. Both experimental monitoring and mathematical modeling confirm that the incorporation of conditional defection and punishment mechanism can jointly stabilize social cooperative behaviors during evolution by transforming the public goods game into a rock-paper-scissors game and therefore solve the problem of public goods dilemma in microbial populations.

## Results

### QS directs tripartite population divergence during evolution

We created the condition of bacterial iterated public goods game (IPG) by growing wild type *P. aeruginosa* PAO1 (WT PAO1) in M9 minimal growth medium [38] containing sodium caseinate (casein) as the sole carbon source. This medium was defined as QS-required medium (QSM) because WT cooperator can digest casein by expressing the QS-controlled extracellular elastase (encoded by the *lasB* gene), while the growth of QS-deficient defector is depended on the cooperation of others [16, 19]. A recent study reported that bacterial cooperation could be stabilized by the fast growth of cooperators in log phase, while the cheating of QS-mutant strain was greatly benefited in stationary phase with abundant public goods [42]. Moreover, there might also be some individuals failed to receipt the QS signals during stationary-phase growth [40, 41]. Therefore, to explore the behavioral diversity in QS-dominated IPG, 1.0–2.0 × 10^7^ CFUs of WT PAO1 were cultured in 1.0 ml of M9-casein (0.5%) medium for 24 h with shaking. Growth of WT PAO1 under this culture condition would rapidly enter into stationary phase yielding a population size of ≈ 1.5 × 10^7^ CFUs. From the second cycle, the culture liquid was diluted (1:10) into the same fresh medium and incubated again for 24 h, and this cycle was continued for 9 days.

Rather than solely targeting the typical *lasR* mutant defectors, here we mainly focused on the public goods production ability of randomly isolated colonies (*n* = 100) from the end of each cycle to evaluate the outcome of IPG. In comparison to the protease-positive cooperators with apparent proteolysis halo on QSM plate after 12 h, a fraction of protease-negative individual with no mutation in *lasR* gene (conditional defector) was detected after the first cycle, and *lasR* mutant defectors began to be identified after 5 cycles (Fig. 1c, d, and Additional file 2: Table S1). Dynamic tripartite equilibrium of cooperator, conditional defector, and mutant defector was finally reached after cycle 8 (Fig. 1c). Interestingly, when the cooperation, conditional defection, and defection strains were picked out and cultured in liquid QSM separately, the population increase and elastase production of conditional defection strain were significantly delayed in the initial (12 h time point) and became comparable to cooperation strain after 24 h, but always higher than those of defection strain (Additional file 3: Figure S2A and B). Accordingly, the growth of conditional defection strain in adenosine medium was significantly slower than cooperation strain at 12 h time point and became comparable to cooperation strain and faster than defection strain during further culture (Additional file 3: Figure S2C). Moreover, when the three kinds of strains were separately applied to the in vitro evolution assay, protease-positive individuals could also emerge and remain in the culture started from pure conditional defection strain (Additional file 3: Figure S2D). These findings implied that the conditional defection strain was capable of restoring the unstable protease-negative phenotype to protease-positive upon nutrient refreshment, and the characteristic of which was more like that of previously identified individual with inactive QS regulation in stationary phase [40, 41]. We speculated that these conditional defectors might actually be a specific state of some cooperators with temporary deficiency in public goods production under nutrient stress condition. This could be supported by the result of whole-genome sequencing, which showed that although 27 unique single nucleotide polymorphism sites causing 13 synonymous and 14 nonsynonymous mutations of 3 genes were detected in conditional defector compared with cooperator (Additional file 4: Dataset S1, PRJNA310762), none of these genes were found to be related with the known QS regulatory profile [32]. Therefore, our results here revealed that in addition to the traditional cooperators and mutant defectors, bacterial QS-dominated social cooperation also created selection for conditional defectors that could coexist with the other two strategists in the IPG by adopting a temporary cheating strategy.

### Environmental nutrient stress contributes to selecting conditional defectors

**Fig. 2:**
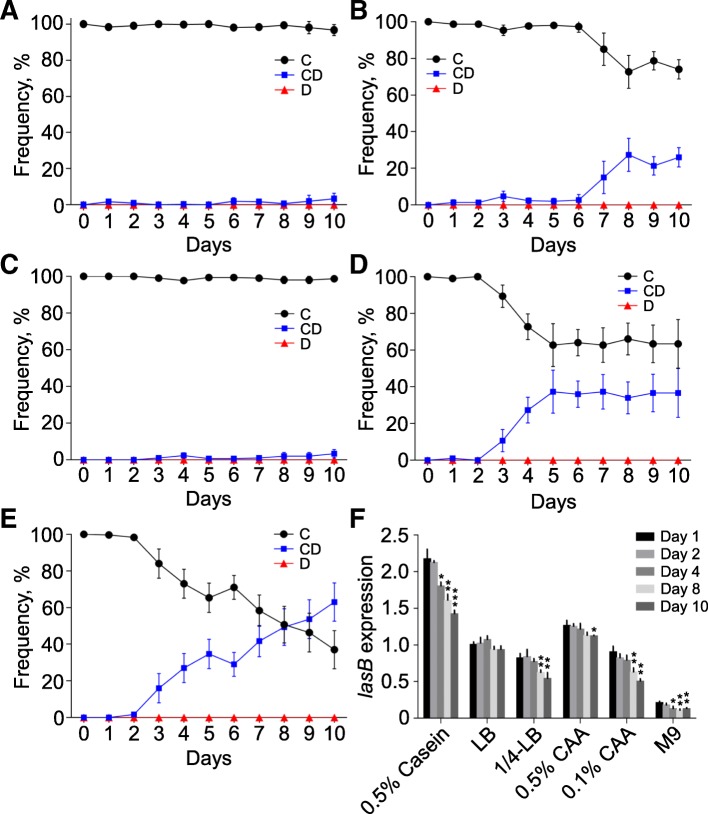
Nutrient deprivation contributes to the emergence of conditional defector. Population divergence of repeatedly subcultured WT PAO1 at 24-h intervals was determined in 1.0 ml of **a** LB, **b** 1/4-LB (4-fold diluted LB), **c** M9-casamino acids (CAA, 0.5%), **d** M9-CAA (0.1%), and **e** blank M9, respectively. **f** Expression of *lasB* gene during the in vitro evolution of *P. aeruginosa* under different culture conditions. The expression values of *lasB* gene in each culture were normalized to that of LB on day 1. Data shown are the mean values ± SD of three independent experiments. Statistical significance by two-tailed unpaired *t* test in comparison to day 1 of each culture is indicated as *P < 0.05; **P < 0.01; ***P < 0.001. C, cooperator; CD, conditional defector; D, defector

Based on the previous finding that bacteria could coordinate the production of QS-controlled public goods upon environmental stimulations [20, 22, 33, 39], we considered that the abundance and composition of nutrient factors might contribute to the emergence of conditional defectors. To test this hypothesis, the population structure of repeatedly subcultured WT PAO1 under the conditions with different nutrient levels and types was monitored. Compared to the tripartite social structure of *P. aeruginosa* formed in QSM (Fig. 1c), no *lasR* mutant defector was detected in rich media or in blank M9 in 10 cycles. Moreover, the level of total public goods (*lasB* expression) in casein medium was significantly higher than that in other media (Additional file 5: Figure S3). These were consistent with previous conclusion that QS-required condition could promote the emergence of QS mutants during evolution [19, 22]. Differently, conditional defectors could still be detected in all cultures and the enrichment of which showed a nutrient level dependent manner (Fig. 2a–e). Furthermore, the expression of *lasB* reduced along with the increasing proportion of conditional defectors (Fig. 2f), and the population sizes were slightly increased when low level of casein or casamino acids was supplemented as the sole carbon source (Additional file 6: Figure S4). These results suggested that bacterial social structure can be readily divided by the enrichment of conditional defectors rather than mutant defectors to cope with the condition of nutrient reduction.

**Fig. 3:**
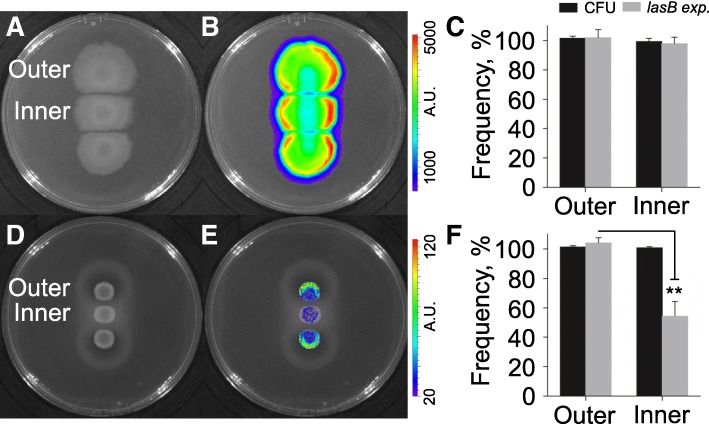
On-plate competition assay. Equal amount of *P. aeruginosa* XEN41 were spotted (5 μl) in triplicate on petri dish in a straight line with a crucial distance of 5.0 mm and cultured for 4 days on LB plate and 3 days on M9-milk plate. **a** Morphology and **b** cell distribution of XEN41 colonies on LB plate. **c** CFU and expression of *lasB* gene in the inner and outer colonies on LB plate. **d** Morphology and **e** cell distribution of XEN41 colonies on M9-milk plate. **f** CFU and expression of *lasB* gene in the inner and outer colonies on M9-milk plate. A.U., absorbance unit. Data shown in panels (**c**) and (**f**) are the percentage CFU (black bars) and *lasB* expression (gray bars) compared with an outer colony and represented by the mean values ± SD of three independent experiments. Statistical significance by two-tailed unpaired *t* test is indicated: **P < 0.01

Subsequently, an on-plate competition assay was developed to further explore the potential strategy shift of cooperators to conditional defectors. PAO1 XEN41 (WT PAO1 constitutively expressing the fluorescent *luxCDABE* cassette) was spotted in triplicate on LB (rich medium) and M9-milk (QSM) plates in a straight line with a distance of 5.0 mm. In this way, the inner colony has relatively less resource available and the growth of which can be greatly influenced by the extracellular products of both outer colonies. The results showed that XEN41 grew fast on LB plate, especially the individuals in the edge of the colony which had more opportunity to obtain abundant nutrient. However, no significant differences of population size and *lasB* expression were detected between the inner and outer colonies (Fig. 3a–c). By contrast, XEN41 grew with no apparent population expansion on QSM plate. Intriguingly, although the population size of the inner colony was similar to that of the outer, the inner colony produced significant lower elastase (*P* = 0.0013, *t* = 8.081, *df* = 4) albeit no *lasR* mutant was detected (Fig. 3d–f). This should be due to the limited nutrient (milk) around the inner colony that had been digested by elastase diffused from the two outer colonies. Therefore, these results directly confirmed that bacteria could dominate the activation of QS system in dependence of environmental nutrient status, and the emergence of conditional defection strategy was due to the decreased/closed QS regulation of some cooperators when investing in public goods production would not bring more benefits.

### Conditional defector can cheat or cooperate in the public goods game

**Fig. 4:**
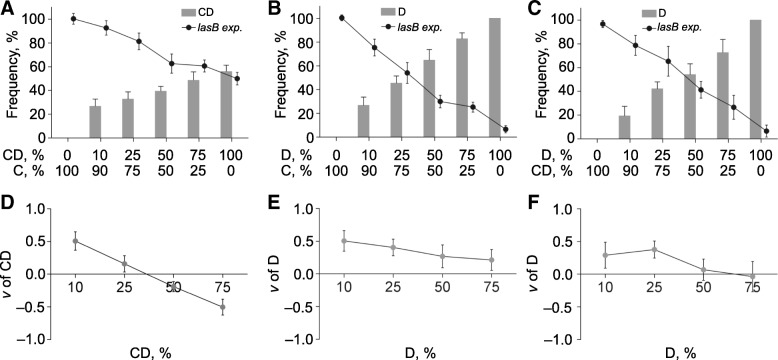
In-tube competition assay. **a** Co-cultures of conditional defector and cooperator (*comlasR*^+^) at different initial ratios were grown in M9-casein (0.5%) broth for 24 h. Shown are the proportion of conditional defector (bars) and the percentage *lasB* expression (line) compared with a *comlasR*^+^ pure culture. **b** Co-cultures of defector (*ΔlasR*) and cooperator at different initial ratios were grown in M9-casein (0.5%) broth for 24 h. Shown are the proportion of defector (bars) and the percentage *lasB* expression (line) compared with a *comlasR*^+^ pure culture. **c** Co-cultures of defector and conditional defector at different initial ratios were grown in M9-casein (0.5%) broth for 24 h. Shown are the proportion of defector (bars) and the percentage *lasB* expression (line) compared with a conditional defector pure culture. **d** Relative fitness of conditional defector in panel **a**. **e** Relative fitness of defector in panel **b**. **f** Relative fitness of defector in panel **c**. Data shown are the mean values ± SD of three independent experiments. C, cooperator; CD, conditional defector; D, defector

We next developed a batch of in-tube competition assay to probe the strategy of conditional defector in the public goods game. To prevent the potential role change of cooperator towards conditional defector which might bias the fitness of conditional defector, the strain *comlasR*^+^ (complemented *lasR* mutant constitutively expressing *lasR*), instead of WT PAO1, was used as cooperator in the competition. The results revealed that conditional defector could behave as mutant defector to defeat cooperator by adopting a cheating strategy; however, the invasion ability of conditional defector to cooperator was lower than that of mutant defector as reflected by their different trends in proportion increasing and in the decreasing of total public goods (*lasB* expression) levels (Fig. 4a, b). Additionally, in comparison to the high relative fitness of mutant defector against cooperator (*v* > 0), the growth advantage of conditional defector was higher than cooperator only when its initial proportion was lower than ~ 25% (Fig. 4d, e). These could be explained by the fixed cheating strategy of mutant defector against cooperator, while conditional defector at high initial proportion was also capable of expressing low level (< 0.5-fold of *comlasR*^+^) of *lasB* (Fig. 4a, b). In this point, conditional defector could invade cooperator only when the competition was started from low conditional defector frequency. Accordingly, when mutant defector was competed with higher proportion (> 50%) of conditional defector, mutant defector could defeat conditional defector by stealing the limited public goods produced by conditional defector (Fig. 4c). However, the growth rate of mutant defector was comparable (*v* ≈ 0) to that of conditional defector when the initial proportion of conditional defector was lower than ~ 25%, because there were no redundant public goods that could be exploited (Fig. 4f). Therefore, these results confirmed that conditional defectors were capable of changing their behaviors according to the strategy and frequency of opponents in the game.

### Conditional defectors benefit from social punishment mechanism

**Fig. 5:**
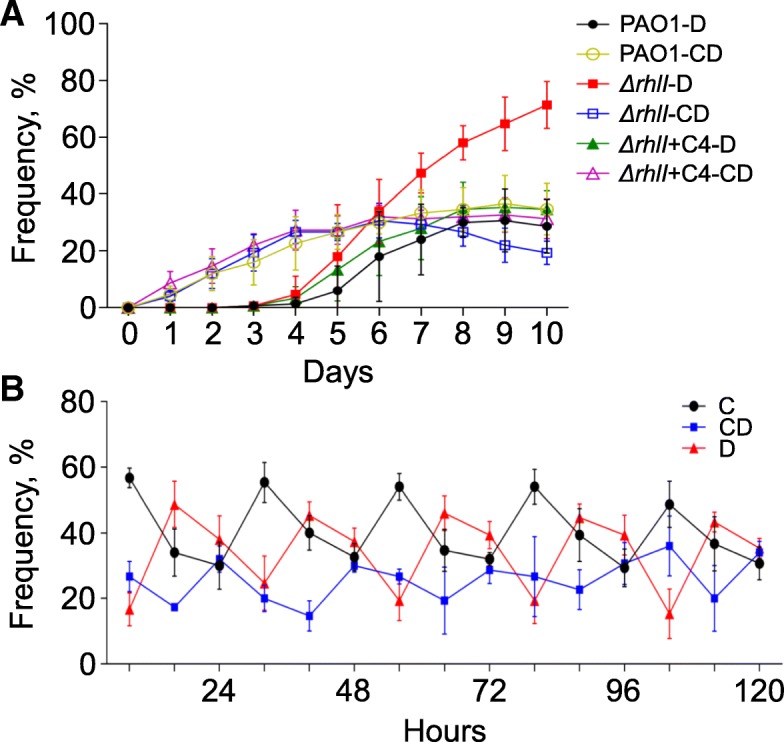
Cooperators, conditional defectors, and defectors can dynamically coexist under the surveillance of social punishment mechanism. **a** Frequencies of conditional defector (hollow symbols) and defector (filled symbols) during in vitro evolution of the punishment deficient strain (*ΔrhlI*) with (triangular symbols) or without (square symbols) the supplementation of C4HSL (C4, 20 μM), and the parental WT PAO1 (round symbols) in M9-casein (0.5%) broth at the end of each cycle. The proportion of cooperator in each culture was not shown in the panel. **b** Frequencies of cooperator, conditional defector, and defector at defined sampling points during in vitro evolution. 100 μl of liquid fractions from the cycle 6 of Fig. 1c were inoculated into M9-casein (0.5%) broth for further repeated subculture at 24-h intervals. Composition of the three strategists in the population was detected at 8 h, 16 h, and 24 h time points after the medium was refreshed in each round. Data shown are the mean values ± SD of three independent experiments. C, cooperator; CD, conditional defector; D, defector

It is reported that the synthesis of cyanide, which is positively regulated by the RhlR-RhlI QS circuit in *P. aeruginosa*, can restrict the prevalence of *lasR* mutant defectors in stationary phase [23, 43]. To explore whether this social punishment mechanism would also harm the fitness of conditional defector, the cyanide-deficient *P. aeruginosa* strain (*ΔrhlI*) with impaired punishment ability was repeatedly subcultured in QSM as described in the in vitro evolution assay above. We found that unlike the emerged *lasR* mutant defectors which finally dominated the culture of *ΔrhlI*, conditional defector was not widespread or even decreased along with the increasing mutant defectors. When the cyanide-production ability of *ΔrhlI* was restored by exogenous C4HSL signal, the relatively stable social structure and population size similar to those in the culture initiated from WT PAO1 were reached again (Fig. 5a and Additional file 7: Figure S5). These data suggested that the intervention of punishment mechanism could guarantee the fitness of cooperator and conditional defector against mutant defector invasion. Furthermore, we were interested to investigate the frequency-dynamic features of the three strategists in each round along with the depletion and re-supplementation of resource supplies. When the well-mixed cooperators, conditional defectors, and mutant defectors were repeatedly subcultured in QSM and the frequency of each player was monitored at different time points of each cycle, we found that the three strategists could coexist in the population with periodically changed frequencies (Fig. 5b). Specifically, the high proportion of cooperators in the initial could be explained by their fast-growth strategy [42] and the strategy shift of some conditional defectors to cooperation when there were abundant nutrient supplies, while the subsequent enrichment of mutant defectors was due to their heritable cheating behavior (Fig. 4 and Additional file 3: Figure S2). Finally, the decreased proportion of mutant defector and enriched conditional defector at the end of each cycle was consistent with the development of social punishment and the survival advantage of conditional defector under the conditions with less resource available (Figs. 2 and 5a).

### Cooperator, conditional defector, and defector can coexist in a rock-paper-scissors dynamics

**Fig. 6:**
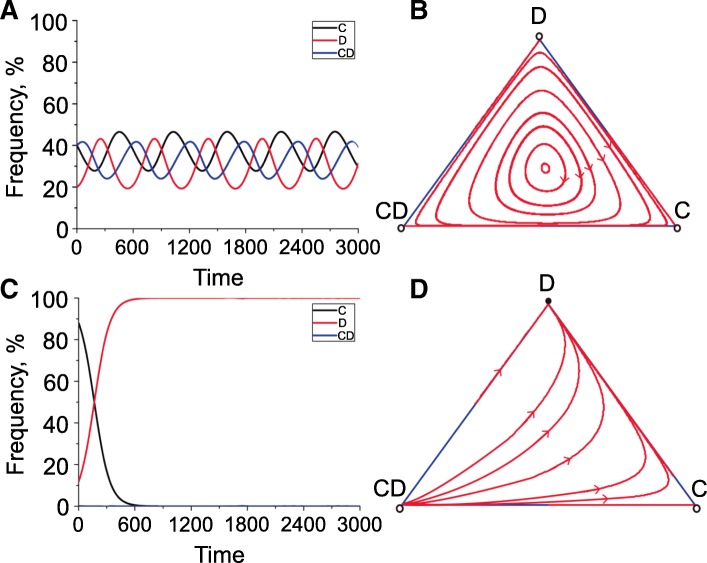
Evolutionary dynamics of cooperation, conditional defection, and defection by mathematical modeling. Mathematical models are conducted under the two scenarios where cooperators are able (**a** and **b**) and unable (**c** and **d**) to exclude defectors, respectively. Panels **a** and **c** depict the time series of frequencies of cooperator (black line), defector (red line), and conditional defector (blue line). Panels **b** and **d** depict the evolutionary trajectories in the simplex, where filled circle represents stable fixed point and open circles represent unstable fixed points. The evolutionary trajectories were obtained through numerical integrations based on the replicator equations in the mathematical model and drawn by using the MATLAB software package (R2014a). Parameters: group size *N* = 5, multiplication factor *r* = 3, contribution cost *c* = 0.3, cost of exclusion *δ* = 0.3, probability of exclusion *p* = 1, transfer rate of conditional defectors *q* = 0, and observation cost *∆* = 0.35. When cooperators are able to exclude defectors, the three strategists can form the periodic oscillations during evolution (**a** and **b**), where the three strategists coexist. In contrast, when cooperators cannot exclude defectors, cooperator and conditional defector are both extinct and defector dominates the whole population finally (**c** and **d**). C, cooperator; CD, conditional defector; D, defector

Based on the experimental characterization above, the evolutionary dynamics of cooperation, conditional defection, and defection in a well-mixed population was further investigated by mathematical modeling in the framework of IPG. Considering the strategy selection of conditional defector could be influenced by environmental factor, we simulated the interaction dynamics of the three strategists by setting the precondition as whether conditional defector would change back to cooperator. Intriguingly, when conditional defector would not transform to cooperator (*q* = 0) and cooperator was able to deterministically exclude defector (*p* = 1), the three strategists could coexist with periodically oscillated frequencies and form cyclically dominated stochastic trajectories (Fig. 6a, b). The outcome of this rock-paper-scissors game in the community could better match the result of experimental monitoring (Fig. 5b). In this condition, conditional defector remained at cheating to overcome cooperator by exploiting the public goods, and this might happen in the microbial populations with abundant public goods (Fig. 4a, d). Defector was superior to conditional defector because conditional defector would theoretically pay a perception cost *∆* to adjust the behavior according to the surrounding environment while defector would contribute nothing. And our mathematical analysis could apply to the experimentally confirmed invasion of defector to conditional defector (Fig. 4c, f). Notably, the key factor to stabilize cooperation in this dynamical interaction system was whether the punishment by cooperator could efficiently reduce the fitness of defector (Fig. 5a). When the exclusion rate (*p*) of cooperator on defector was set as 0.8, which meant that a defector could not be deterministically excluded by cooperator, we found that cooperator had the highest growth advantage only in the initial stage and each strategy (especially defector) would have the opportunity to dominate the population after 500 rounds (Additional file 8: Figure S6). We further confirmed the role of punishment in the game by removing the punishment ability of cooperator. In this way, the strategy of cooperator was accordant to the *rhlI* null cooperators which could cooperate but failed to exclude defector (Fig. 5a). Accordingly, full defection was always the only stable state no matter what initial condition was considered (Fig. 6c, d), thereby confirming the necessity of punishment for the stabilization of social structure.

We then checked the evolutionary dynamics of cooperation, conditional defection, and defection when a portion of conditional defectors would change to cooperate (*q* = 0.1). This precondition was true only when the production of public goods was beneficial to the fitness of cooperator, for example, in the log-phase growth of bacterial population with less public goods and abundant nutrient supply. We found that a low exclusion rate (*p* = 0.2) of cooperator on defector would rapidly lead to the prevalence of defector, and cooperators would dominate the population for a longer time when *p* was set as 0.8. Moreover, when *p* = 1, although the frequency of defector was greatly suppressed, cooperator and conditional defector would alternatively dominate the game because a portion of conditional defector would transform to cooperate when the frequency of cooperator was low (Additional file 9: Figure S7). However, the deduced evolutionary dynamics with parameter *p* = 1 might not happen in real-life IPG within this context, because the cyanide-mediated punishment mechanism in *P. aeruginosa* would be peaked in stationary phase along with the abolishment of RhlR expression [43, 44]. Therefore, our mathematical prediction here also provided a theoretical explanation for the prosperity of bacterial cooperators during log-phase growth, either by using the underdeveloped RhlRI-dependent cyanide production ability or by the fast-growth strategy to prevent energy waste and defector exploitation.

## Discussion

The discovery and elucidation of QS system in microbial populations have significantly contributed to the development of sociomicrobiology, particularly on the issues of cooperation and defection. Understanding the dynamics of cell-cell interactions in pathogenic bacteria has important biological and clinical significance [3, 17, 45, 46]. By characterizing the behavioral composition and their evolutionary dynamics in bacterial QS-dominated cooperative interaction, here we introduce the conditional defector, which is a QS-inactive state of cooperator and can stabilize the coexistence of cooperative and cheating behaviors in a rock-paper-scissors dynamics.

Prior work by Köhler et al. identified several clinical *P. aeruginosa* isolates with remarkable diversity of QS phenotypes in causing chronic lung infection of cystic fibrosis patients [45]. Although *lasR* mutants were found to be the most prevalent, there were also a portion of isolates with intact *lasR* gene but showed decreased QS ability. Heurlier et al. also identified a set of *lasR*-intact but elastase-negative strains in the initial rounds of repeatedly subcultured *P. aeruginosa* [47]. However, the role of these strains in the population was not characterized. In the present study, no mutation site which may block elastase production is identified in the genome of conditional defector (Additional file 4: Dataset S1). This provides a molecular basis for the unstable protease-negative phenotype of conditional defectors, which can readily revert to protease-positive under resource-abundant condition by modulating the function of QS system (Fig. 3 and Additional file 3: Figure S2). These genetic and phonotypical evidences indicate that the role change of cooperator to conditional defector in bacterial game should be due to the reprogramming of intracellular regulation in response to nutrient reduction rather than solely depending on mutagenesis. In *P. aeruginosa*, the function of QS system can be repressed by at least five proteins (RsmA, QscR, MvaT, RpoN, and RsaL) [27]. Specifically, the small global regulatory protein RsaL can bind with the central QS regulator LasR to the bidirectional promoter region of *rsaL-lasI* in stationary phase and contributes to the homeostasis of QS system by coordinating the level of QS signal [48, 49]. QscR is a homolog of LasR and RhlR and controls the timing of QS-related gene expression also by reducing the level of LasI-produced signal [50]. Moreover, the alternative sigma factor RpoN (σ^54^) may negatively regulate the activation of QS system through GacA-RsmA signaling [51]. Therefore, the inactivation of bacterial QS system is multiply controlled by the intricate regulatory networks under different growth conditions, and the intracellular protein binding and stripping processes may provide an underlying mechanistic explanation for the maintenance and inter-conversion of cooperator and conditional defector upon environmental change.

By monitoring the evolutionary dynamics of cooperative and cheating behaviors in bacterial IPG, we find that bacterial social structure can be readily diverged by the strategy shift of some WT individuals from cooperation to conditional defection (Figs. 1c and 2). The subsequent emergence and enrichment of mutant defectors are the consequence of natural selection for individuals that can better adapt to the conditions with less resource and increased social conflict for public goods [19, 34, 52]. In this trend, conditional defection can be considered as a preferred strategy which enables the renaissance of social cooperation in better conditions. By contrast, cooperators can prevent the prevalence of mutant defectors by using cyanide-mediated punishment mechanism [23]. The persistence of these defectors in the community indicates that the punishment mechanism may only function in a certain period, and this is accordant to the expression trend of cyanide which is significantly increased from log phase and peaked in stationary phase [44]. Moreover, although the enriched conditional defectors with inactive QS regulation may also weaken the social punishment, mutant defector will not prosper during further culture because of the limited public goods in the community. Finally, cooperators, conditional defectors, and defectors can dynamically coexist under the joint effect of nutrient renewal and punishment mechanism. Further mechanistic study concerning the regulation of cyanide-mediated cytotoxicity/detoxification and social selection of conditional defector in clinical *P. aeruginosa* populations will facilitate our further understanding of the evolutionary dynamics of cooperation and the pathogenesis of pseudomonal chronic diseases.

Bacteria are proficient in solving the toxin-mediated social conflict and in maximizing the population fitness under antibiotic stress by forming the rock-paper-scissors dynamics [26, 53, 54]. Our mathematical modeling analysis uncovers a novel application of these dynamics in the category of QS-dominated cooperative interaction. When the strategy of conditional defector is restricted to cheating, which means a low resource supply and accumulated public goods in the initial, cooperators, conditional defectors, and defectors can coexist with periodically oscillated frequencies in a rock-paper-scissors dynamics under the surveillance of punishment mechanism (Fig. 6). Accordingly, increasing the nutrient level will allow the transformation of conditional defector to cooperator, and thus, cooperators have more opportunity to dominate the interaction. It is predictable that this model will also slip to the rock-paper-scissors game when the nutrient refresh cycle was prolonged, because the conditional defector will finally stay in cheating when investing in public goods fails to bring more benefits in a single round of culture (Fig. 2 and Additional file 9: Figure S7). This evolution-determined behavioral heterogeneity enables QS microbes to reasonably allocate the limited resources in a “time-sharing” trend and increases the elasticity of population in adaptation to various adverse environments [55–57]. Such kind of behavioral heterogeneity induced by conditional strategy also exists in the societies of advanced species [58] and has been demonstrated to play an important role in the evolution of cooperation in complicated game systems [59–62]. Thus, our work also contributes to understanding the evolution of cooperation in human society.

## Conclusions

Public goods dilemma is ubiquitous in microbial populations and therefore arouses the burgeoning development of sociomicrobiology. Our current work highlights the importance of behavioral heterogeneity for stabilizing social cooperation in microbial communities, provides an answer for the outstanding question regarding the coexistence of cooperation and defection during evolution, and may open up a new avenue for further characterization of microbial cell-cell communications.

## Methods

### Bacterial strains and media

Wild type *P. aeruginosa* PAO1 and isogeneic *lasR* and *rhlI* mutants used in this study were previously preserved in the laboratory. Bacteria from a single colony were grown in LB broth/agar or in M9 medium [38] supplemented with different nutrient factors.

### In vitro evolution assay

WT PAO1 cells from a single colony were first inoculated into LB broth and cultured at 37 °C with shaking (220 rpm) for 12 h. Bacterial cells were then harvested, and about 1.0–2.0 × 10^7^ cells were separately inoculated into 1.0 ml of QSM [M9–0.5% (*w*/*v*) casein (Sigma)], blank M9 broth, LB broth, 4-fold diluted LB broth (1/4-LB), M9 broth supplemented with 0.5% or 0.1% casamino acids (CAA, Sigma) and cultured at 37 °C for 24 h with shaking (220 rpm). From the second cycle, the culture liquids were diluted (1:10) into accordant fresh medium and incubated again for 24 h, and this cycle was continued for 9 days. Moreover, 1.0–2.0 × 10^7^ CFUs of *rhlI* mutant (signal deficient strain in the *rhl* QS system) cells were also repeatedly subcultured in 1.0 ml of QSM with/without exogenous C4HSL (20 μM) signal molecule (Sigma). Subsequently, 50 μl of culture liquids from the in vitro evolution assays at the end of each cycle were diluted by sterile saline solution and transferred onto LB plate or QSM plate [M9-casein/skim milk (0.5%)] and cultured for 8–16 h. A fraction of culture liquid (100 μl) from the end of cycle 6 of in vitro evolution of WT PAO1 in QSM were transferred to another set of repeated subculture in 1.0 ml of QSM at 24-h intervals, and 50 μl of culture liquids at the 8 h, 16 h, and 24 h time points of each cycle were also spread on LB or QSM plates.

### Phenotypic and genetic identification

A total of 100 colonies were randomly picked out from each LB plate above and transferred onto QSM plate to identify their protease production activity according to the size of proteolysis halo around each colony. The strains with poor protease production activity (in comparison to the WT phenotype) at 12 h time point were conducted for further DNA sequencing of the full region of *lasR* gene (from 220 bp upstream to 419 bp downstream of *lasR* gene) using primer pair 5′-ACGCTGCGGTCTATTGTTA-3′ and 5′-ATCTCGCCCAGCAGTTTT-3′ to screen the *lasR* mutant strain. PCR amplification was performed using Q5® high-fidelity DNA polymerase (New England Biolabs). The *lasR* intact strains with or without apparent proteolysis halo on QSM plate from day 3 of the in vitro evolution assay, and the identified *lasR* mutant strain was shortly (4–8 h) enriched in LB broth. Subsequently, the three kinds of strains were separately transferred into 1.0 ml of M9-casein medium and cultured for 12 h, 24 h, and 24 h followed by CFU enumeration on LB agar and elastin-Congo red (Sigma)-based protease production assay as described previously [63]. Moreover, the three kinds of strains were also separately conducted for the in vitro evolution assay in M9-casein (0.5%) broth to determine the frequency of protease-positive strains at the end of each cycle.

### Whole-genome sequencing

Genomic DNAs of protease-positive strain and protease-negative strain with no mutation in *lasR* gene (conditional defection strain) from the in vitro evolution assay were harvested and sequenced by using the Illumina HiSeq 2500-PE150 platform (Personal Biotechnology Co., Ltd., China). The generated sequencing data had been deposited in the NCBI database under the accession number PRJNA407264. The high-quality reads were mapped to the model genome sequence of PAO1 (GenBank accession number: AE004091) with Bowtie2 [64]. Duplicates were then removed with Picard (http://broadinstitute.github.io/picard/). Pileup files were built with SAMtools [65], and SNPs detection was performed using VarScan2 [66] with thresholds of a minimum of 8 supporting variant reads and variant allele frequencies of at least 1%.

### On-plate competition experiments

Equal amount (1.0 × 10^6^ CFUs) of PAO1 XEN41 (Calpier, Inc.) were spotted (5 μl) in triplicate on LB plate and modified M9 plate (Na_2_HPO_4_, KH_2_PO_4_, and NaCl were replaced by 5.0 g/L of KH_2_PO_4_ and 1.0 g/L of NaCl) supplemented with 0.5% (*w*/*v*) skim milk powder (Bio-Rad) in a straight line with a crucial distance of 5.0 mm. The growth and distribution of bacterial cells on the plates were assessed by checking the bioluminescence using the IVIS XRII imaging system (Calpier, Inc.). Subsequently, the inner and outer colonies were separately scraped and diluted in 1.0 ml of sterile saline solution. The appropriately diluted cell resuspension solutions were spread on LB plates for CFU enumeration and on QSM plates to determine the protease production phenotype and *lasR* gene sequence of the strains as described above.

### In-tube competition experiments

*P. aeruginosa lasR* mutant complemented with the plasmid pAK1900 constitutively expressing *lasR* gene (*comlasR*^+^) [67], conditional defection strain, and *ΔlasR* were prepared in pair (1.0 × 10^7^ CFUs in total) at different ratios and cultured in 1.0 ml of QSM for 24 h. The final percentage of each strain was determined by spreading 50 μl of diluted culture liquids on M9-milk plate. The *comlasR*^+^ strain always showed a proteolysis halo around the colony after 12 h, and the conditional defection strain and *ΔlasR* with no apparent proteolysis halo were further discriminated by PCR to amplify a partial region of *lasR* gene [34]. The relative fitness (*v*) of conditional defection strain or *ΔlasR* in corresponding co-culture was then calculated by comparing the initial (*x*_0_) and final frequencies (*x*_1_) using the modified equation *v* = log_10_[*x*_1_(1 − *x*_0_)/ *x*_0_(1 − *x*_1_)] as described elsewhere [16]. In this study, *v* > 0 indicates one player grows faster than the opponent, while *v* < 0 indicates one player grows slower than the opponent.

### Quantitative PCR

Bacterial cells from days 1, 2, 4, 8 to 10 of another independent complete set of in vitro evolution assay of WT PAO1 and from the competition experiments above were harvested. Total bacterial RNA was isolated by RNAprep pure Cell/Bacteria Kit (TIANGEN) according to the manufacturer’s instructions. The expression of *lasB* gene was determined by quantitative PCR using QuantiTect SYBR Green RT-PCR Kit (QIAGEN) and CFX Connect™ Real-Time PCR Detection System (Bio-Rad). 16S rRNA was used as the internal reference, and the relative expression was calculated using the 2^-*ΔΔCT*^ method [34].

### Statistical analyses

Data analysis and statistical tests were performed by using Graphpad Prism version 7.0 (San Diego, CA, USA). Mean values of standard deviation (SD) were compared by using two-tailed unpaired *t* test.

### Mathematical modeling

Mathematical models were developed in the framework of iterated public goods game to predict the evolutionary dynamics of cooperation and defection in a well-mixed population as previously introduced for microbial games [13, 15, 24], since our experiments were performed in well-mixed culture where each individual was assumed to respond to the mean of the population.

*N* individuals are randomly chosen to form a group, and they can choose to cooperate or defect. C (cooperator) will contribute *c* to produce public goods, while D (defector) would contribute nothing. For the punishment of C on D, we assume that each C should pay a cost *δ* to exclude (kill) D [25], and it can successfully exclude a D from the group with a probability *p*. The probability that a defector is not excluded from a group having *N*_*C*_ cooperators is ${\left(1-p\right)}^{N_C}$. All individuals’ contributions are summed up and multiplied by an enhancement factor *r* and then equally distributed among group members.

We first consider the replicator dynamics for C and D in a large well-mixed population, with frequencies *x* and 1 − *x*, respectively. Thus, the replicator equation [24] is:

$$ \dot{x}=x\left(1-x\right)\left({P}_C-{P}_D\right). $$

Then the average payoffs for cooperators and defectors can be respectively given by:

$$ {P}_C=\sum \limits_{N_C=0}^{N-1}\left(\genfrac{}{}{0pt}{}{N-1}{N_C}\right){x}^{N_C}{\left(1-x\right)}^{N-{N}_C-1}\sum \limits_{N_W=0}^{N-{N}_C-1}\left(\begin{array}{c}N-{N}_C-1\\ {}{N}_W\end{array}\right){\left[{\left(1-p\right)}^{N_C+1}\right]}^{N_W}{\left[1-{\left(1-p\right)}^{N_C+1}\right]}^{N-{N}_C-1-{N}_W}{\pi}_C, $$

$$ {P}_D=\sum \limits_{N_C=0}^{N-1}\left(\genfrac{}{}{0pt}{}{N-1}{N_C}\right){x}^{N_C}{\left(1-x\right)}^{N-{N}_C-1}\sum \limits_{N_W=0}^{N-{N}_C-1}\left(\begin{array}{c}N-{N}_C-1\\ {}{N}_W\end{array}\right){\left[{\left(1-p\right)}^{N_C}\right]}^{N_W}{\left[1-{\left(1-p\right)}^{N_C}\right]}^{N-{N}_C-1-{N}_W}{\pi}_D, $$

where *N*_*C*_ represents the number of C in the group, *p* denotes the probability that one D is excluded successfully by one C in the group, *N*_*W*_ represents the number of D who have not been excluded by the C, *π*_*D*_ and *π*_*C*_ represent the payoffs of a D and a C obtained from the group, respectively. In addition, we have:

$$ {\pi}_D={\left(1-p\right)}^{N_C}\frac{rc{N}_C}{N_C+{N}_W+1}, $$

$$ {\pi}_C=\frac{rc\left({N}_C+1\right)}{N_C+{N}_W+1}-c-\delta . $$

Subsequently, conditional defector (CD) is introduced into the game. We assume that CD as a conditional strategy will pay a perception cost *∆* to adjust the behavior according to the surrounding environment. For simplicity, we suppose that a fraction *q* of CD will transform to cooperate and others (1 − *q*) remained cheating during the evolutionary process. In addition, each *C* should pay a cost *δ* to successfully exclude a *D* with a probability *p*, and the probability that a *D* is not excluded from a group having *N*_*C*_ cooperators is ${\left(1-p\right)}^{N_C}$. We consider that individuals can adopt one of the three strategies in a large well-mixed population with frequencies of *x*, *y*, *z*, respectively. Thus, we have *x*, *y*, *z* ≥ 0 and *x* + *y* + *z* = 1. We denote the average payoffs for three strategists by *P*_*I*_ with *I* = *C*, *D*, or *CD*, respectively. According to the standard replicator dynamics, the replicator equations are given by:

$$ \dot{x}=x\left({P}_C-\overline{P}\right), $$

$$ \dot{y}=y\left({P}_D-\overline{P}\right), $$

$$ \dot{z}=z\left({P}_{CD}-\overline{P}\right), $$

where $\overline{P}=x{P}_C+y{P}_D+z{P}_{CD}$ describes the average payoff of the population. The average payoffs for each strategy can be respectively given as:

$$ {P}_C=\sum \limits_{N_C=0}^{N-1}\sum \limits_{N_D=0}^{N-1-{N}_C}\sum \limits_{N_{CD}=0}^{N-1-{N}_C-{N}_D}\frac{\left(N-1\right)!}{N_C!{N}_D!{N}_{CD}!}{\left(x+ qz\right)}^{N_C}{y}^{N_D}{\left[\left(1-q\right)z\right]}^{N_{CD}}\sum \limits_{N_W=0}^{N_D}\left(\begin{array}{c}{N}_D\\ {}{N}_W\end{array}\right){\left[{\left(1-p\right)}^{N_C+1}\right]}^{N_W}{\left[1-{\left(1-p\right)}^{N_C+1}\right]}^{N_D-{N}_W}{\pi}_C, $$

$$ {P}_D=\sum \limits_{N_C=0}^{N-1}\sum \limits_{N_D=0}^{N-1-{N}_C}\sum \limits_{N_{CD}=0}^{N-1-{N}_C-{N}_D}\frac{\left(N-1\right)!}{N_C!{N}_D!{N}_{CD}!}{\left(x+ qz\right)}^{N_C}{y}^{N_D}{\left[\left(1-q\right)z\right]}^{N_{CD}}\sum \limits_{N_W=0}^{N_D}\left(\begin{array}{c}{N}_D\\ {}{N}_W\end{array}\right){\left[{\left(1-p\right)}^{N_C}\right]}^{N_W}{\left[1-{\left(1-p\right)}^{N_C}\right]}^{N_D-{N}_W}{\pi}_D, $$

and

$$ {P}_{CD}=\sum \limits_{N_C=0}^{N-1}\sum \limits_{N_D=0}^{N-1-{N}_C}\sum \limits_{N_{CD}=0}^{N-1-{N}_C-{N}_D}\frac{\left(N-1\right)!}{N_C!{N}_D!{N}_{CD}!}{\left(x+ qz\right)}^{N_C}{y}^{N_D}{\left[\left(1-q\right)z\right]}^{N_{CD}}\sum \limits_{N_W=0}^{N_D}\left(\begin{array}{c}{N}_D\\ {}{N}_W\end{array}\right){\left[{\left(1-p\right)}^{\left(1-q\right){N}_C+q\left({N}_C+1\right)}\right]}^{N_W}{\left[1-{\left(1-p\right)}^{\left(1-q\right){N}_C+q\left({N}_C+1\right)}\right]}^{N_D-{N}_W}{\pi}_{CD}, $$

where *N*_*C*_, *N*_*D*_, and *N*_*CD*_ respectively represent the numbers of C, D, and CD in the group; *N*_*W*_ represents the number of D who have not been excluded by C; and *π*_*D*_, *π*_*CD*_, and *π*_*C*_ represent the payoffs of a D, a CD, and a C obtain from the group, respectively. Accordingly, $\frac{\left(N-1\right)!}{N_C!{N}_D!{N}_{CD}!}$ and $$ \left(\begin{array}{c}{N}_D\\ {}{N}_W\end{array}\right) $$represent the multinomial and binomial coefficients, respectively, and $\frac{\left(N-1\right)!}{N_C!{N}_D!{N}_{CD}!}{\left(x+ qz\right)}^{N_C}{y}^{N_D}{\left[\left(1-q\right)z\right]}^{N_{CD}}$ describes the probability of finding the (*N* − 1) co-players with *N*_*C*_ cooperators, *N*_*D*_ defectors, and *N*_*CD*_ conditional defectors. In addition, we have:

$$ {\pi}_D={\left(1-p\right)}^{N_C}\frac{rc{N}_C}{N_C+{N}_{CD}+{N}_W+1}, $$

$$ {\pi}_{CD}=\left(1-q\right)\frac{rc{N}_C}{N_C+{N}_{CD}+{N}_W+1}+q\left[\frac{rc\left({N}_C+1\right)}{N_C+{N}_{CD}+{N}_W+1}-c-\delta \right]-\Delta , $$

and

$$ {\pi}_C=\frac{rc\left({N}_C+1\right)}{N_C+{N}_{CD}+{N}_W+1}-c-\delta . $$

Please see Additional file 10 for more detailed analysis of mathematical models.

## Additional files

Additional file 1: **Figure S1.** The gradient of selection in dependence on the fraction of cooperators for different exclusion probabilities in infinite populations. Stable equilibria are described by solid circles, while unstable equilibria are described by open circles. Arrows indicate the expected direction of evolution. Cooperation is more favored by natural selection when the arrow points to the right. (A) When the exclusion probability is significantly small, full defection is the only stable equilibrium in the population. (B) With a larger exclusion probability, a coordination game with full cooperation and full defection as the two stable equilibria appears. (C) When the exclusion probability is further increased, full cooperation is the only stable equilibrium. The values of exclusion probability are (A) *p* = 0.1; (B) *p* = 0.6; (C) *p* = 0.8. Other parameters: *N* = 5, *r* = 3, *c* = 0.3, and *δ* = 0.3. (PDF 262 kb)

Additional file 2: **Table S1.** Characterization of *lasR* mutants during the in vitro evolution of wild type *P. aeruginosa* in 1.0 ml of M9-casein (0.5%) broth. (PDF 200 kb)

Additional file 3: **Figure S2.** Phenotypic identification of cooperator, conditional defector, and defector. (A) Growth curves of mono-cultured cooperation strain, conditional defection strain, and defection strain in 1.0 ml of M9-casein (0.5%) broth. Cultures were started from equal number of cells (1 × 10^5^ CFUs, which was diluted from OD_600_ = 0.005). Data are mean values ± SD (log_10_ of CFUs) of three individual experiments. (B) Percentage elastase production of conditional defection and defection strains compared with a cooperation strain pure culture. The liquid supernatants (10 μl) of each strain from the experiment in panel (A) were conducted for elastin-Congo red based protease production assay. All the absorbance values at 495 nm were normalized to that of a cooperation strain at the 12 h time point. Data shown are the mean values ± SD of three independent experiments. Statistical significance by two-tailed unpaired *t*-test is indicated: *P < 0.05, **P < 0.01, ***P < 0.001. (C) Growth of conditional defection strain and defection strain compared with a cooperation strain in M9-adenosine broth. Equal amount of cooperation strain, defection strains and conditional defection strains from the in vitro evolution assay were cultured in liquid M9-adenosine (0.5%) followed by CFU enumeration at different time points. All the CFU values were normalized to that of a cooperation strain at the 12 h time point. Data shown are the mean values ± SD of three independent experiments. Statistical significance by two-tailed unpaired *t*-test is indicated: *P < 0.05, **P < 0.01, ***P < 0.001. (D) Percentage of protease-positive individuals in repeatedly (24-h intervals) subcultured cooperation strain, conditional defection strain, and defection strain strains, respectively. The frequencies of protease production individuals in each culture were determined at the end of each cycle. Data shown are the mean values ± SD of three independent experiments. C, cooperator. CD, conditional defector. D, defector. (PDF 125 kb)

Additional file 4: **Dataset S1.** Single nucleotide polymorphism sites of conditional defection strain different from that of cooperation strain. (XLSX 10 kb)

Additional file 5: **Figure S3.** Comparison of *lasB* expression under different culture conditions. The expression values of *lasB* gene in each culture were normalized to that of LB on day 1. Data shown are the mean values ± SD of three independent experiments. Statistical significance by two-tailed unpaired *t* test in comparison to 0.5% casein of each column is indicated as: ***P < 0.001. CAA, casamino acids. (PDF 112 kb)

Additional file 6: **Figure S4.** Population sizes of repeatedly subcultured *P. aeruginosa* in 1.0 ml of different media at 24-h intervals. (A) M9-casein (0.5%). (B) Blank M9. (C) LB. (D) 1/4-LB. (E) M9-casamino acids (CAA, 0.5%). (F) M9-CAA (0.1%). Data shown are the mean values ± SD (log_10_ of CFUs) of three independent experiments. (PDF 175 kb)

Additional file 7: **Figure S5.** Population sizes of the punishment deficient strain (*ΔrhlI*) and parental WT PAO1 in 1.0 ml of M9-casein (0.5%) broth at the end of each cycle. The punishment mechanism of *ΔrhlI* can be repaired by the supplementation of C4HSL (C4, 20 μM). Data shown are the mean values ± SD (log_10_ of CFUs) of three independent experiments. (PDF 164 kb)

Additional file 8: **Figure S6.** Evolutionary dynamics of cooperation, conditional defection, and defection in the simplex with moderately high exclusion probability. Panel (A) depicts the time series of frequencies of cooperator (black lines), defector (red lines), and conditional defector (blue lines). Panels (B) depicts the evolutionary trajectories in the simplex. Parameters: group size *N* = 5, multiplication factor *r* = 3, contribution cost *c* = 0.3, cost of exclusion *δ* = 0.3, probability of exclusion *p* = 0.8, transfer rate of conditional defectors *q* = 0, and observation cost *∆* = 0.35. C, cooperator. CD, conditional defector. D, defector. (PDF 303 kb)

Additional file 9: **Figure S7.** Evolutionary dynamics of cooperation, conditional defection, and defection in the simplex for different exclusion probabilities (*p* = 0.2 for top row, *p* = 0.8 for middle row, and *p* = 1 for bottom row). Panels (A), (C), and (E) depict the time series of frequencies of cooperator (black lines), defector (red lines), and conditional defector (blue lines). Panels (B), (D), and (F) depict the evolutionary trajectories in the simplex, where filled circle represents stable fixed point and open circles represent unstable fixed points. When the exclusion probability is relatively small, the system converges to the full defection state finally (A and B), indicating that defectors dominate the whole population. Whereas when the exclusion probability is relatively high, the periodic oscillations among the three strategists appear (C–F), leading to the stabilization of cooperators in the pool. Other parameters: *N* = 5, *r* = 3, *c* = 0.3, *δ* = 0.3, *∆* = 0.35, and *q* = 0.1. C, cooperator. CD, conditional defector. D, defector. (PDF 423 kb)

Additional file 10: Analysis of mathematical models. (PDF 359 kb)
