# Supplementary material for: Behavioral heterogeneity in quorum sensing can stabilize social cooperation in microbial populations
Source: BMC Biol. 2019 Mar 6;17:20. doi: 10.1186/s12915-019-0639-3 (PMC6889464; doi:10.1186/s12915-019-0639-3)
Supplement: Supplementary file 1 — Figure S1. The gradient of selection in dependence on the fraction of cooperators for different exclusion probabilities in infinite populations. Stable equilibria are described by solid circles, while unstable equilibria are described by open circles. Arrows indicate the expected direction of evolution. Cooperation is more favored by natural selection when the arrow points to the right. (A) When the exclusion probability is significantly small, full defection is the only stable equilibrium in the population. (B) With a larger exclusion probability, a coordination game with full cooperation and full defection as the two stable equilibria appears. (C) When the exclusion probability is further increased, full cooperation is the only stable equilibrium. The values of exclusion probability are (A) p = 0.1; (B) p = 0.6; (C) p = 0.8. Other parameters: N = 5, r = 3, c = 0.3, and δ = 0.3. (PDF 262 kb) [file 12915_2019_639_MOESM1_ESM.pdf]

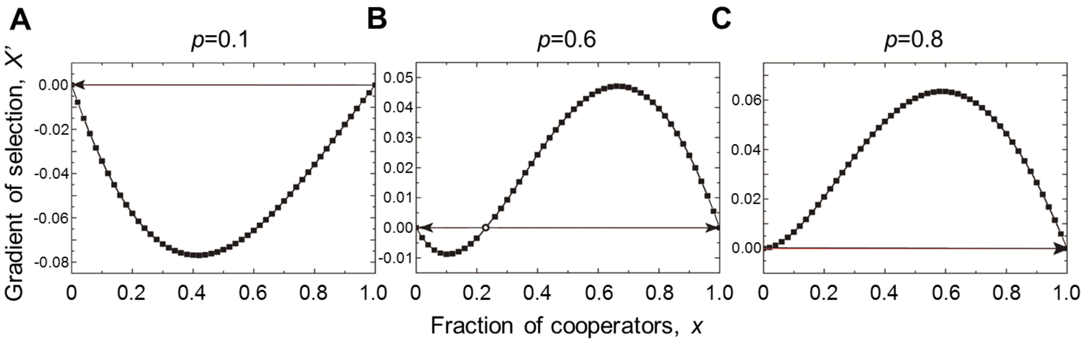

Additional file 1: Figure S1. The gradient of selection in dependence on the fraction of cooperators for different exclusion probabilities in infinite populations. Stable equilibria are described by solid circles, while unstable equilibria are described by open circles. Arrows indicate the expected direction of evolution. Cooperation is more favored by natural selection when the arrow points to the right. (**A**) When the exclusion probability is significantly small, full defection is the only stable equilibrium in the population. (**B**) With a larger exclusion probability, a coordination game with full cooperation and full defection as the two stable equilibria appears. (**C**) When the exclusion probability is further increased, full cooperation is the only stable equilibrium. The values of exclusion probability are (**A**) $p = 0.1$; (**B**) $p = 0.6$; (**C**) $p = 0.8$. Other parameters: $N = 5, r = 3, c = 0.3$, and $\delta = 0.3$.
