# Supplementary material for: Behavioral heterogeneity in quorum sensing can stabilize social cooperation in microbial populations
Source: BMC Biol. 2019 Mar 6;17:20. doi: 10.1186/s12915-019-0639-3 (PMC6889464; doi:10.1186/s12915-019-0639-3)
Supplement: Supplementary file 2 — Table S1. Characterization of lasR mutants during the in vitro evolution of wild type P. aeruginosa in 1.0 ml of M9-casein (0.5%) broth. (PDF 200 kb) [file 12915_2019_639_MOESM2_ESM.pdf]

Additional file 2: Table S1. Characterization of *lasR* mutants during the *in vitro* evolution of wild-type *P. aeruginosa* in 1.0 ml of M9-casein (0.5%) broth.

Variants	Cycle	Mutation[1]	Change[2]	Protease[3]	Adenosine[4]
lasR1	5	A→T (+643)	Ile → Phe	–	–
lasR2	9	G→A (+541)	Glu → Lys	–	–
ΔlasR	N/A	Deletion	N/A	–	–
WT PAO1	N/A	None	N/A	++	++

N/A, not applicable.

[1]Sites of nucleotide mutation relative to translational start site of the *P. aeruginosa* PAO1 *lasR* gene.

[2]Amino acid changes relative to the LasR protein sequence of *P. aeruginosa* PAO1.

[3]'+' indicates the presence and '–' indicates the absence of a proteolysis halo on M9-casein plate.

[4]'+', positive growth (wild-type phenotype); '–', negative growth (defined *lasR* mutant phenotype).
