# Supplementary material for: Behavioral heterogeneity in quorum sensing can stabilize social cooperation in microbial populations
Source: BMC Biol. 2019 Mar 6;17:20. doi: 10.1186/s12915-019-0639-3 (PMC6889464; doi:10.1186/s12915-019-0639-3)
Supplement: Supplementary file 3 — Figure S2. Phenotypic identification of cooperator, conditional defector, and defector. (A) Growth curves of mono-cultured cooperation strain, conditional defection strain, and defection strain in 1.0 ml of M9-casein (0.5%) broth. Cultures were started from equal number of cells (1 × 10^5^ CFUs, which was diluted from OD600 = 0.005). Data are mean values ± SD (log10 of CFUs) of three individual experiments. (B) Percentage elastase production of conditional defection and defection strains compared with a cooperation strain pure culture. The liquid supernatants (10 μl) of each strain from the experiment in panel (A) were conducted for elastin-Congo red based protease production assay. All the absorbance values at 495 nm were normalized to that of a cooperation strain at the 12 h time point. Data shown are the mean values ± SD of three independent experiments. Statistical significance by two-tailed unpaired t-test is indicated: *P < 0.05, **P < 0.01, ***P < 0.001. (C) Growth of conditional defection strain and defection strain compared with a cooperation strain in M9-adenosine broth. Equal amount of cooperation strain, defection strains and conditional defection strains from the in vitro evolution assay were cultured in liquid M9-adenosine (0.5%) followed by CFU enumeration at different time points. All the CFU values were normalized to that of a cooperation strain at the 12 h time point. Data shown are the mean values ± SD of three independent experiments. Statistical significance by two-tailed unpaired t-test is indicated: *P < 0.05, **P < 0.01, ***P < 0.001. (D) Percentage of protease-positive individuals in repeatedly (24-h intervals) subcultured cooperation strain, conditional defection strain, and defection strain strains, respectively. The frequencies of protease production individuals in each culture were determined at the end of each cycle. Data shown are the mean values ± SD of three independent experiments. C, cooperator. CD, conditional defector. D, defector. [file 12915_2019_639_MOESM3_ESM.pdf]

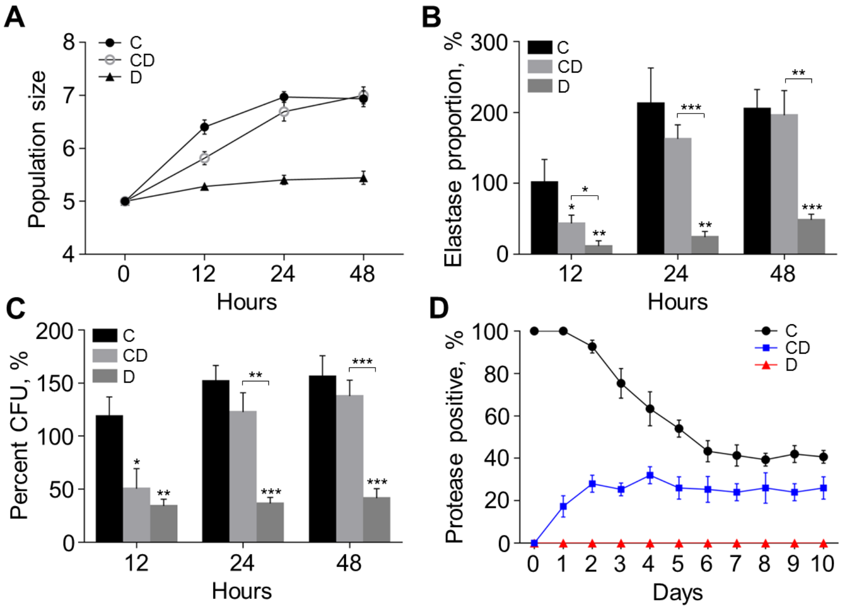

Additional file 3: Figure S2. Phenotypic identification of cooperator, conditional defector, and defector. (**A**) Growth curves of mono-cultured cooperation strain, conditional defection strain, and defection strain in 1.0 ml of M9-casein (0.5%) broth. Cultures were started from equal number of cells (1×10^5 CFUs, which was diluted from $OD_{600} = 0.005$). Data are mean values ± SD ($\log_{10}$ of CFUs) of three individual experiments. (**B**) Percentage elastase production of conditional defection and defection strains compared with a cooperation strain pure culture. The liquid supernatants (10 μl) of each strain from the experiment in panel (**A**) were conducted for elastin-Congo red based protease production assay. All the absorbance values at 495 nm were normalized to that of a cooperation strain at the 12 h time-point. Data shown are the mean values ± SD of three independent experiments. Statistical significance by two-tailed unpaired *t*-test is indicated: *$P < 0.05$, **$P < 0.01$, ***$P < 0.001$. (**C**) Growth of conditional defection strain and defection strain compared with a cooperation strain in M9-adenosine broth. Equal amount of cooperation strain, defection strains and conditional defection strains from the *in vitro* evolution assay were cultured in liquid M9-adenosine (0.5%) followed by CFU enumeration at different time-points. All the CFU values were normalized to that of a cooperation strain at the 12 h time-point. Data shown are the mean values ± SD of three independent experiments. Statistical significance by two-tailed unpaired *t*-test is indicated: *$P < 0.05$, **$P < 0.01$, ***$P < 0.001$. (**D**) Percentage of protease-positive individuals in repeatedly (24-h intervals) subcultured cooperation strain, conditional defection strain, and defection strain strains, respectively. The frequencies of protease production individuals in each culture were determined at the end of each cycle. Data shown are the mean values ± SD of three independent experiments. C, cooperator. CD, conditional defector. D, defector.
