# Supplementary material for: Behavioral heterogeneity in quorum sensing can stabilize social cooperation in microbial populations
Source: BMC Biol. 2019 Mar 6;17:20. doi: 10.1186/s12915-019-0639-3 (PMC6889464; doi:10.1186/s12915-019-0639-3)
Supplement: Supplementary file 5 — Figure S3. Comparison of lasB expression under different culture conditions. The expression values of lasB gene in each culture were normalized to that of LB on day 1. Data shown are the mean values ± SD of three independent experiments. Statistical significance by two-tailed unpaired t test in comparison to 0.5% casein of each column is indicated as: ***P < 0.001. CAA, casamino acids. (PDF 112 kb) [file 12915_2019_639_MOESM5_ESM.pdf]

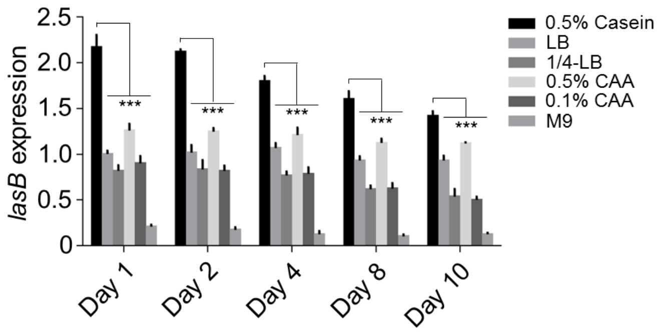

Additional file 5: Figure S3. Comparison of *lasB* expression under different culture conditions. The expression values of *lasB* gene in each culture were normalized to that of LB on day 1. Data shown are the mean values ± SD of three independent experiments. Statistical significance by two-tailed unpaired *t*-test in comparison to 0.5% casein of each column is indicated as: ***$P < 0.001$. CAA, casamino acids.
