# Supplementary material for: Behavioral heterogeneity in quorum sensing can stabilize social cooperation in microbial populations
Source: BMC Biol. 2019 Mar 6;17:20. doi: 10.1186/s12915-019-0639-3 (PMC6889464; doi:10.1186/s12915-019-0639-3)
Supplement: Supplementary file 6 — Figure S4. Population sizes of repeatedly subcultured P. aeruginosa in 1.0 ml of different media at 24-h intervals. (A) M9-casein (0.5%). (B) Blank M9. (C) LB. (D) 1/4-LB. (E) M9-casamino acids (CAA, 0.5%). (F) M9-CAA (0.1%). Data shown are the mean values ± SD (log10 of CFUs) of three independent experiments. (PDF 175 kb) [file 12915_2019_639_MOESM6_ESM.pdf]

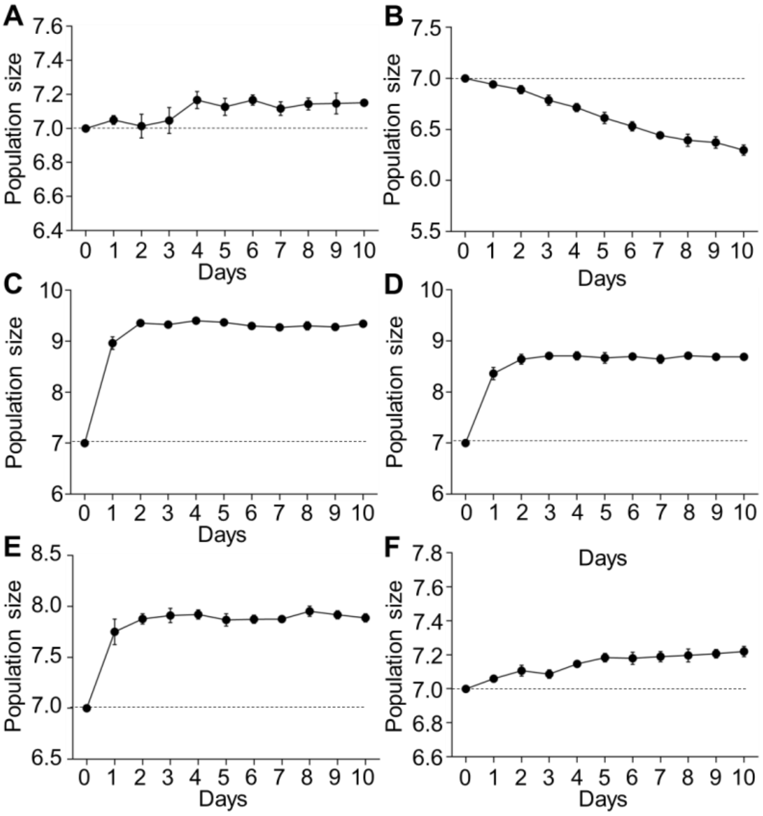

Additional file 6: Figure S4. Population sizes of repeatedly subcultured *P. aeruginosa* in 1.0 ml of different media at 24-h intervals. (**A**) M9-casein (0.5%). (**B**) Blank M9. (**C**) LB. (**D**) 1/4-LB. (**E**) M9-casamino acids (CAA, 0.5%). (**F**) M9-CAA (0.1%). Data shown are the mean values ± SD (log_{10} of CFUs) of three independent experiments.
