# Supplementary material for: Behavioral heterogeneity in quorum sensing can stabilize social cooperation in microbial populations
Source: BMC Biol. 2019 Mar 6;17:20. doi: 10.1186/s12915-019-0639-3 (PMC6889464; doi:10.1186/s12915-019-0639-3)
Supplement: Supplementary file 7 — Figure S5. Population sizes of the punishment deficient strain (ΔrhlI) and parental WT PAO1 in 1.0 ml of M9-casein (0.5%) broth at the end of each cycle. The punishment mechanism of ΔrhlI can be repaired by the supplementation of C4HSL (C4, 20 μM). Data shown are the mean values ± SD (log10 of CFUs) of three independent experiments. (PDF 164 kb) [file 12915_2019_639_MOESM7_ESM.pdf]

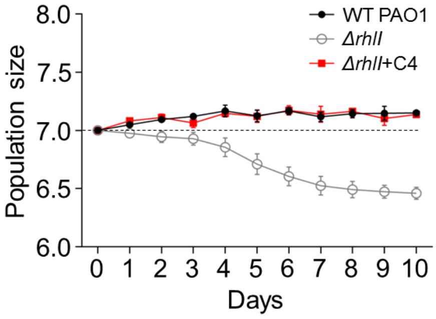

Additional file 7: Figure S5. Population sizes of the punishment deficient strain (*ΔrhlI*) and parental WT PAO1 in 1.0 ml of M9-casein (0.5%) broth at the end of each cycle. The punishment mechanism of *ΔrhlI* can be repaired by the supplementation of C4HSL (C4, 20 μM). Data shown are the mean values ± SD ($\log_{10}$ of CFUs) of three independent experiments.
