# Supplementary material for: Behavioral heterogeneity in quorum sensing can stabilize social cooperation in microbial populations
Source: BMC Biol. 2019 Mar 6;17:20. doi: 10.1186/s12915-019-0639-3 (PMC6889464; doi:10.1186/s12915-019-0639-3)
Supplement: Supplementary file 8 — Figure S6. Evolutionary dynamics of cooperation, conditional defection, and defection in the simplex with moderately high exclusion probability. Panel (A) depicts the time series of frequencies of cooperator (black lines), defector (red lines), and conditional defector (blue lines). Panels (B) depicts the evolutionary trajectories in the simplex. Parameters: group size N = 5, multiplication factor r = 3, contribution cost c = 0.3, cost of exclusion δ = 0.3, probability of exclusion p = 0.8, transfer rate of conditional defectors q = 0, and observation cost ∆ = 0.35. C, cooperator. CD, conditional defector. D, defector. (PDF 303 kb) [file 12915_2019_639_MOESM8_ESM.pdf]

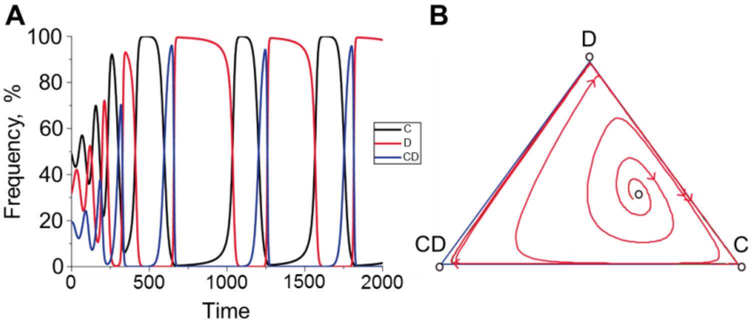

Additional file 8: Figure S6. Evolutionary dynamics of cooperation, conditional defection, and defection in the simplex with moderately high exclusion probability. Panel (**A**) depicts the time series of frequencies of cooperator (black lines), defector (red lines), and conditional defector (blue lines). Panels (**B**) depicts the evolutionary trajectories in the simplex. Parameters: group size $N = 5$, multiplication factor $r = 3$, contribution cost $c = 0.3$, cost of exclusion $\delta = 0.3$, probability of exclusion $p = 0.8$, transfer rate of conditional defectors q=0, and observation cost $\Delta = 0.35$. C, cooperator. CD, conditional defector. D, defector.
