# Supplementary material for: Behavioral heterogeneity in quorum sensing can stabilize social cooperation in microbial populations
Source: BMC Biol. 2019 Mar 6;17:20. doi: 10.1186/s12915-019-0639-3 (PMC6889464; doi:10.1186/s12915-019-0639-3)
Supplement: Supplementary file 9 — Figure S7. Evolutionary dynamics of cooperation, conditional defection, and defection in the simplex for different exclusion probabilities (p = 0.2 for top row, p = 0.8 for middle row, and p = 1 for bottom row). Panels (A), (C), and (E) depict the time series of frequencies of cooperator (black lines), defector (red lines), and conditional defector (blue lines). Panels (B), (D), and (F) depict the evolutionary trajectories in the simplex, where filled circle represents stable fixed point and open circles represent unstable fixed points. When the exclusion probability is relatively small, the system converges to the full defection state finally (A and B), indicating that defectors dominate the whole population. Whereas when the exclusion probability is relatively high, the periodic oscillations among the three strategists appear (C–F), leading to the stabilization of cooperators in the pool. Other parameters: N = 5, r = 3, c = 0.3, δ = 0.3, ∆ = 0.35, and q = 0.1. C, cooperator. CD, conditional defector. D, defector. (PDF 423 kb) [file 12915_2019_639_MOESM9_ESM.pdf]

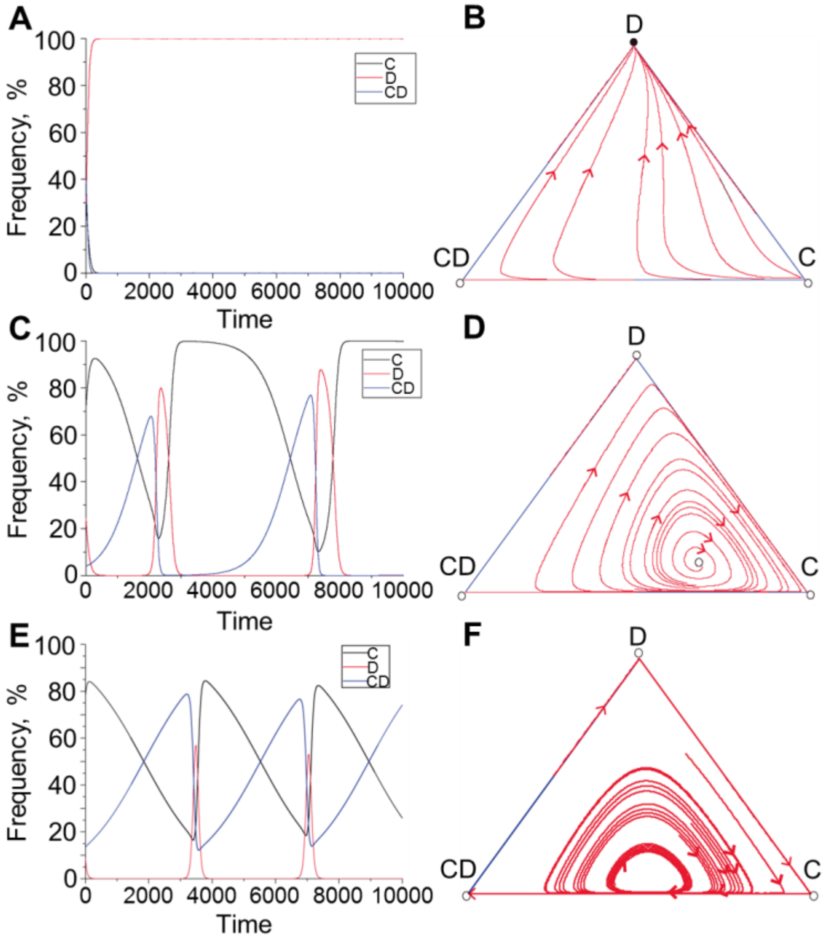

Additional file 9: Figure S7. Evolutionary dynamics of cooperation, conditional defection, and defection in the simplex for different exclusion probabilities ($p = 0.2$ for top row, $p = 0.8$ for middle row, and $p = 1$ for bottom row). Panels (**A**), (**C**), and (**E**) depict the time series of frequencies of cooperator (black lines), defector (red lines), and conditional defector (blue lines). Panels (**B**), (**D**), and (**F**) depict the evolutionary trajectories in the simplex, where filled circle represents stable fixed point and open circles represent unstable fixed points. When the exclusion probability is relatively small, the system converges to the full defection state finally (**A** and **B**), indicating that defectors dominate the whole population. Whereas when the exclusion probability is relatively high, the periodic oscillations among the three strategists appear (**C**–**F**), leading to the stabilization of cooperators in the pool. Other parameters: $N = 5$, $r = 3$, $c = 0.3$, $\delta = 0.3$, $\Delta = 0.35$, and $q = 0.1$. C, cooperator. CD, conditional defector. D, defector.
