# Supplementary material for: Behavioral heterogeneity in quorum sensing can stabilize social cooperation in microbial populations
Source: BMC Biol. 2019 Mar 6;17:20. doi: 10.1186/s12915-019-0639-3 (PMC6889464; doi:10.1186/s12915-019-0639-3)
Supplement: Supplementary file 10 — Analysis of mathematical models. (PDF 359 kb) [file 12915_2019_639_MOESM10_ESM.pdf]

Additional file 10: Analysis of mathematical models

Based on the expressions of mathematical modeling in the Methods section, here we provide the corresponding theoretical analysis as follows for Fig. 6.

Scenario 1: cooperators cannot exclude defectors. In this case, we have

$$\pi_D = \frac{rcN_C}{N},$$

$$\pi_{\mathrm{CD}} = (1-q)\frac{rcN_C}{N} + q[\frac{rc(N_C+1)}{N} - c] - \Delta$$

$$= \frac{rcN_C}{N} + q\frac{rc}{N} - qc - \Delta,$$

and

$$\pi_C = \frac{rc(N_C+1)}{N} - c.$$

Accordingly, the average payoffs for cooperator (C), conditional defector (CD), and defector (D) are respectively given by

$$P_C = \frac{rcx(N-1)+rc}{N} - c,$$

$$P_D = \frac{rcx(N-1)}{N},$$

and

$$P_{CD} = \frac{rcx(N-1)}{N} + q\frac{rc}{N} - qc - \Delta.$$

Then we discuss the equilibrium points of the system. By substituting $x = 1 - y - z$, we can get

$$\dot{y} = y[(1-y)(P_D - P_C) - z(P_{CD} - P_C)],$$
$$\dot{z} = z[(1-z)(P_{CD} - P_C) - y(P_D - P_C)],$$

where $P_D - P_C = c - \frac{rc}{N}$ and $P_D - P_{CD} = qc\left(1 - \frac{r}{N}\right) + \Delta$. For $r < N$, we have $P_D > P_C$ and $P_D > P_{CD}$. Therefore there is no interior fixed point and the system only has three corner fixed points, namely, (x, y, z) = (1, 0, 0), (0, 1, 0), and (0, 0, 1), respectively in the C-D-CD simplex. Furthermore, D has an evolutionary advantage over C and CD. Accordingly, the system will ultimately end up in the full D state, which is consistent with numerical calculations in Fig. 6c, d.

Scenario 2: cooperators are able to exclude defectors. As shown in Fig. 6a, b, we have the exclusion probability $p = 1$. We then have that the probability of finding, among the $N-1$ other players in the sample, $S-1$ co-players sharing the public goods is

$$\binom{N-1}{S-1}(1-y)^{S-1}y^{N-S}.$$

Furthermore, the probability that there are k cooperators and $S-k-1$ conditional defectors is

$$\binom{S-1}{k}\left(\frac{x}{x+z}\right)^k\left(\frac{z}{x+z}\right)^{S-1-k}.$$

Hence the expected payoff of a CD in a group of S players is

$$\sum_{k=0}^{S-1}\binom{S-1}{k}\left(\frac{x}{x+z}\right)^{k}\left(\frac{z}{x+z}\right)^{S-1-k}\left(\frac{rck}{S}-\Delta\right)=\frac{rc}{S}(S-1)\frac{x}{x+z}-\Delta.$$

Thus,

$$P_{CD}=\frac{rcx}{x+z}\sum_{S=1}^{N}\binom{N-1}{S-1}(1-y)^{S-1}y^{N-S}\left(1-\frac{1}{S}\right)-\Delta=\frac{rcx}{1-y}\left[1-\frac{1-y^{N}}{N(1-y)}\right]-\Delta.$$

And the expected payoff of a C in a group of S players is

$$\sum_{k=0}^{S-1}\binom{S-1}{k}\left(\frac{x}{x+z}\right)^{k}\left(\frac{z}{x+z}\right)^{S-1-k}\left[\frac{rc(k+1)}{S}-c-\delta\right]=\frac{rc}{S}(S-1)\frac{x}{x+z}+\frac{rc}{S}-c-\delta.$$

As a result, we have

$$P_{C}=\sum_{S=1}^{N}\binom{N-1}{S-1}(1-y)^{S-1}y^{N-S}\left[\frac{rc}{S}(S-1)\frac{x}{x+z}+\frac{rc}{S}-c-\delta\right]=\frac{rc}{1-y}\left[x+\frac{z(1-y^{N})}{N(1-y)}\right]-c-\delta,$$

$$P_{D}=0,$$

and

$$P_{CD}-P_{\mathrm{C}}=(c+\delta-\Delta)-\frac{rc}{1-y}\frac{(1-y^{N})}{N}.$$

We emphasize that the following inequalities $0<rc-c-\delta$ and $c+\delta-\Delta-\frac{rc}{N}>0$ hold, such that the members in a group where all C who exclude D are better off than D, but CD are better off than members in a group of C since the latter does not exclude the former. Furthermore, we define the function $F(y)=P_{CD}-P_{C}=(c+\delta-\Delta)-\frac{rc}{1-y}\frac{(1-y^{N})}{N}$, and thus $F(y)=0$ being the equilibrium condition. We consider the function $G(y)=(1-y)F(y)$ which has the same roots as $F(y)$ in $(0,1)$. We can get that $G(0)=(c+\delta-\Delta)-\frac{rc}{N}$ and $G(1)=0$. And $G'(y)=(1-y)F'(y)-F(y)$ and $G'(1)=rc-c-\delta+\Delta$. Furthermore, $G''(y)=rc(N-1)y^{N-2}>0$ for $N\geq 2$. Therefore, when $\frac{rc}{N}<c+\delta-\Delta<rc$, there might exist an interior fixed point. In addition, there are three corner fixed points, namely, (x, y, z) = (1, 0, 0), (0, 1, 0), and (0, 0, 1), respectively.

We first study the stability of the three corner fixed points. We respectively define

$$h(y,z)=y[(1-y)(P_{D}-P_{C})-z(P_{CD}-P_{C})],$$

and

$$g(y,z)=z[(1-z)(P_{CD}-P_{C})-y(P_{D}-P_{C})].$$

Accordingly, the Jacobian matrix can be given as

$$J=\begin{bmatrix}\frac{\partial h(y,z)}{\partial y} & \frac{\partial h(y,z)}{\partial z}\\ \frac{\partial g(y,z)}{\partial y} & \frac{\partial g(y,z)}{\partial z}\end{bmatrix},$$

where

$$\frac{\partial h}{\partial y}(y,z)=[(1-y)(P_{D}-P_{C})-z(P_{CD}-P_{C})]+y[-(P_{D}-P_{C})+(1-y)\frac{\partial}{\partial y}(P_{D}-P_{C})-z\frac{\partial}{\partial y}(P_{CD}-P_{C})],$$

$$\frac{\partial h}{\partial z}(y,z)=y\left[(1-y)\frac{\partial}{\partial z}(P_{D}-P_{C})-(P_{CD}-P_{C})-z\frac{\partial}{\partial z}(P_{CD}-P_{C})\right],$$

$$\frac{\partial g}{\partial y}(y,z) = z\left[(1-z)\frac{\partial}{\partial y}(P_{CD}-P_C)-(P_D-P_C)-y\frac{\partial}{\partial y}(P_D-P_C)\right],$$

and

$$\frac{\partial g}{\partial z}(y,z) = [(1-z)(P_{CD}-P_C)-y(P_D-P_C)]+z[-(P_{CD}-P_C)+(1-z)\frac{\partial}{\partial z}(P_{CD}-P_C)-$$

$$y\frac{\partial}{\partial z}(P_D-P_C)].$$

(1) For the corner fixed point (1, 0, 0), we have

$$\frac{\partial h}{\partial y}(0,0) = P_D-P_C = -(rc-c-\delta) < 0,$$

$$\frac{\partial h}{\partial z}(0,0) = 0,$$

$$\frac{\partial g}{\partial y}(0,0) = 0,$$

and

$$\frac{\partial g}{\partial z}(0,0) = P_{CD}-P_C = c+\delta-\Delta-\frac{rc}{N}.$$

As a result, the Jacobian is

$$J = \begin{bmatrix} -(rc-c-\delta) & 0 \\ 0 & c+\delta-\Delta-\frac{rc}{N} \end{bmatrix}.$$

Therefore, the fixed point is unstable since $c+\delta-\Delta-\frac{rc}{N} > 0$.

(2) For the corner fixed point (0, 1, 0), we have

$$\frac{\partial h}{\partial y}(1,0) = -(P_D-P_C) = rc-c-\delta > 0,$$

$$\frac{\partial h}{\partial z}(1,0) = -(P_{CD}-P_C) = -(c+\delta-\Delta-rc),$$

$$\frac{\partial g}{\partial y}(1,0) = 0,$$

and

$$\frac{\partial g}{\partial z}(1,0) = P_{CD}-P_D = -\Delta < 0.$$

As a result, the Jacobian is

$$J = \begin{bmatrix} rc-c-\delta & -(c+\delta-\Delta-rc) \\ 0 & -\Delta \end{bmatrix},$$

thus the fixed point is a saddle node and unstable.

(3) For the corner fixed (0, 0, 1), we have

$$\frac{\partial h}{\partial y}(0,1) = (P_D-P_{CD}) = \Delta > 0,$$

$$\frac{\partial h}{\partial z}(0,1) = 0,$$

$$\frac{\partial g}{\partial y}(0,1) = -(P_D-P_C) = \frac{rc}{N}-c-\delta,$$

and

$$\frac{\partial g}{\partial z}(0,1) = -(P_{CD} - P_C) = \Delta + \frac{rc}{N} - c - \delta.$$

As a result, the Jacobian is

$$J = \begin{bmatrix} \Delta & 0 \\ \frac{rc}{N} - c - \delta & \Delta + \frac{rc}{N} - c - \delta \end{bmatrix}.$$

Thus, the fixed point is unstable since $\Delta > 0$.

We further study the dynamics at the interior fixed point if it is present in the simplex. To do that, we introduce a new variable $f = \frac{x}{x+z}$, representing the fraction of C among individuals actually sharing the public goods. Thus we have

$$\dot{f} = \frac{\dot{x}(z+x) - x(\dot{z}+\dot{x})}{(z+x)^2} = -f(1-f)(P_{CD} - P_C).$$

On the other hand, $\dot{y} = y(P_D - \bar{P})$, where $\bar{P} = yP_D + zP_{CD} + xP_C = -x(P_{CD} - P_C) + (1-y)(P_{CD} - P_D) + P_D$, resulting in that

$$\dot{y} = y[x(P_{CD} - P_C) - (1-y)(P_{CD} - P_D)] = y(1-y)[f(c+\delta-\Delta-rc) + \Delta].$$

Thus we have

$$\begin{cases} \dot{f} = -f(1-f)[(c+\delta-\Delta) - \frac{rc}{1-y}\frac{(1-y^N)}{N}]. \\ \dot{y} = y(1-y)[f(c+\delta-\Delta-rc) + \Delta]. \end{cases}$$

The separability of the factors allows us to write

$$\frac{dy}{df} = \frac{y(1-y)}{(c+\delta-\Delta) - \frac{rc}{1-y}\frac{(1-y^N)}{N}} \frac{f(c+\delta-\Delta-rc)+\Delta}{-f(1-f)},$$

such that

$$\int \frac{(c+\delta-\Delta) - \frac{rc}{1-y}\frac{(1-y^N)}{N}}{y(1-y)} dy = \int \frac{f(c+\delta-\Delta-rc)+\Delta}{-f(1-f)} df.$$

The integral of the right–hand side is

$$(c+\delta-\Delta-rc)\, log(1-f) - \Delta[log(f) - log(1-f)].$$

The integral of the left–hand side is

$$(c+\delta-\Delta)[log(y) - log(1-y)] - \frac{rc}{N}\int \frac{1-y^N}{y(1-y)^2} dy,$$

where

$$\frac{rc}{N}\int \frac{1-y^N}{y(1-y)^2} dy$$

$$= \frac{rc}{N}\left[log(y) - log(y-1) - \frac{1}{y-1}\right] - \frac{rc}{N}\left[\frac{1}{y-1} - \log(1-y) + \sum_{t=2}^{N-1}\binom{N-1}{t}(-1)^t \frac{(1-y)^{t-1}}{t-1}\right]$$

$$+ Const.$$

In this way, we identify the constant of motion

$$H(f,y) = (c+\delta-\Delta-rc)\, log(1-f) - \Delta[log(f) - log(1-f)] + (c+\delta-\Delta)[log(y) - log(1-y)] - \frac{rc}{N}\left[log(y) - log(y-1) - \frac{1}{y-1}\right] + \frac{rc}{N}\left[\frac{1}{y-1} - \log(1-y) + \sum_{t=2}^{N-1}\binom{N-1}{t}(-1)^t \frac{(1-y)^{t-1}}{t-1}\right].$$

Therefore, we have

$$\dot{H} = \frac{\partial H}{\partial f}\dot{f} + \frac{\partial H}{\partial y}\dot{y} = 0.$$

Thus the fixed point in the simplex is a center surrounded by closed and periodic orbits, as confirmed by numerical calculations in Fig. 6b.

Finally, we show the evolutionary dynamics of the three strategists for different *p* values by numerical calculations, as plotted in Additional file 9: Figure S7. We find that for a small exclusion probability, full D is the only stable state in the system (Additional file 9: Figure S7A, B). It suggests that when the punishment mechanism does not work effectively, D can still dominate the whole population no matter whether the CD is present or not. While for a high exclusion probability, we find that periodic oscillations happen among the three strategies, indicating that C, CD and D coexist when an effective social punishment is available (Additional file 9: Figure S7C, D, E, and F).
